# Females Display Greater Disease Severity in a Mouse Model of Vitiligo

**DOI:** 10.1111/pcmr.70134

**Published:** 2026-11-05

**Authors:** Kaitlyn G. O'Donnell, Zhussipbek Mukhatayev, Hanin Abuzer, Rohan S. Shivde, Sriram Saravanan Shanmugavelandy, Octavio K. Bartos, Ellis Kang, Emilia R. Dellacecca, Dinesh Jaishankar, Surya Pandey, Hee Kap Kang, Ziyou Ren, I. Caroline Le Poole

**Affiliations:** ^1^ Indiana University School of Medicine Indianapolis Indiana USA; ^2^ Department of Dermatology, Microbiology and Immunology, Feinberg School of Medicine Northwestern University Chicago Illinois USA; ^3^ Mayo Clinic College of Medicine and Science, Mayo Clinic Rochester Minnesota USA; ^4^ Department of Surgery, Feinberg School of Medicine Northwestern University Chicago Illinois USA; ^5^ Department of Hematology and Oncology, Feinberg School of Medicine Northwestern University Chicago Illinois USA; ^6^ Department of Pediatrics, Lurie Children's Hospital Northwestern University Chicago Illinois USA

**Keywords:** autoimmune, depigmentation, heat shock protein 70 (HSP70), IFN‐γ, mouse model, sex bias, vitiligo

## Abstract

Vitiligo is an autoimmune disease characterized by progressive depigmentation of the skin due to T cell‐mediated destruction of melanocytes. Disease onset varies but usually initiates during adolescence. Sex differences in disease development and therapeutic outcomes have not been explored. We previously associated stress induced HSP70i with immune activation in depigmentation and showed that HSP70i_Q435A_ can tolerize dendritic cells (DCs) to prevent cutaneous recruitment and activation of T cells. Among male and female vitiligo‐prone mice, we investigated the long‐term efficacy of HSP70i_Q435A_ plasmid DNA treatment introduced by biolistic administration at varying stages of vitiligo development. Mice were followed up to 46 weeks of age. Female mice exhibited 1.89‐fold greater depigmentation than males overall, while both responded proportionally to treatment with effects lasting throughout. This sex difference was associated with significantly elevated IFN‐γ secretion by melanocyte‐reactive T cells among female mice. Humoral responses to the protein remained readily detectable at end point regardless of treatment interval. These findings also suggest greater benefit from earlier intervention.

## Research Highlights

- HSP70i_Q435A_ introduced by biolistic administration of DNA reduced depigmentation in a large cohort of vitiligo‐prone mice long term.
- HSP70i_Q435A_ administration on vitiligo‐affected skin was associated with marked antibody titers to the stress protein that remained detectable after 40 weeks.
- Sex of the animals impacts depigmentation, with vitiligo developing more aggressively in affected females.
- Female depigmentation was associated with more T cells secreting IFN‐γ in response to cognate peptide.
- Differential abundance of Tregs and Type 2 T cells likewise differentiates the 2 sexes.

## Graphical abstract

We compared depigmentation in vitiligo‐prone, h3TA2 mice among males and females, and explored the effects of treatment with *HSP70i*_*Q435A*_ plasmid DNA, delivered by biolistic administration. Mice shown from each cohort exemplify increased depigmentation among female mice and the treatment benefit of HSP70i_Q435A_.

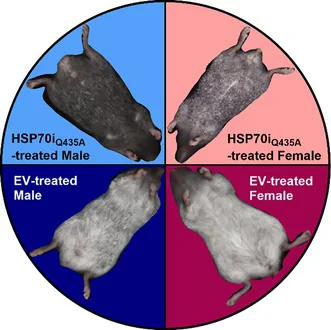

## 1 Introduction

Vitiligo is an autoimmune disease presenting as progressive depigmentation of the skin, hair, and mucosa due to T cell‐mediated melanocyte destruction (Ogg et al. 1998). Patients with vitiligo present with depigmented lesions on the face, hands, ankles, groin, and areas affected by friction or trauma (Gauthier et al. 2003; Stiegler and Brickley 2021). Disease onset can occur at any age with approximately half of cases starting before age 20 (Henning et al. 2020; Silverberg 2014). Vitiligo affects approximately 0.5% to 2% of the world's population (Kruger and Schallreuter 2012) with varied prevalence among different global regions (Zhang et al. 2016).

Although depigmentation is more apparent in individuals with darker skin tones, vitiligo affects individuals of all racial and ethnic groups. Gradual depigmentation causes many patients to experience stigmatization, especially in regions where vitiligo may be confused with leprosy (Millington and Levell 2007). Individuals with vitiligo are more likely to experience depression, anxiety, and social phobia (Ezzedine et al. 2021). Younger age is a risk factor for these comorbidities, with age < 30 years associated with greater psychosocial burden (Ezzedine et al. 2021).

A sex difference in disease prevalence or severity has not been firmly established, yet some studies support more frequent disease development among females (Cedercreutz et al. 2010; Zhang et al. 2016). A higher rate of diagnosis of vitiligo reported in females (Gupta and Economopoulos 2025) might be attributable to greater perceived stress among women, while several autoimmune diseases are more frequent among women (Henning et al. 2020), pointing to shared etiology. The different tendency to seek medical help for social and cosmetic concerns may reflect actual differences in disease development (Patil et al. 2014; Shajil et al. 2006).

Our current understanding of the immunopathological basis of disease has facilitated the development of molecular‐targeted therapies for vitiligo. Melanin formation involves the generation of reactive oxygen species (ROS), causing oxidative stress (Xie et al. 2016). Cellular stress (Boissy and Manga 2004) can provoke melanocytes, especially when derived from vitiligo patients, to secrete inducible HSP70 (HSP70i) (Mosenson et al. 2012). This stress protein protects cells from undergoing apoptosis (Mosser et al. 2000), yet upon secretion, HSP70i activates dendritic cells (DCs) to enhance antigen processing and presentation, leading to accelerated depigmentation in mice (Mosenson et al. 2013). DCs recruit melanocyte‐specific, self‐reactive CD8^+^ T cells to the skin when triggered by inducible HSP70 (Badri et al. 1993; Mosenson et al. 2013; Wankowicz‐Kalinska et al. 2003). Cytotoxic T cells then mediate melanocyte cell death and secrete IFN‐γ and other proinflammatory cytokines to recruit additional inflammatory cells to the site (van den Boorn et al. 2009). This understanding of vitiligo etiology has broadened the options for treatment.

The identification of HSP70i as a protein mediating the initiation of depigmentation has instigated further study into a modified version of the protein as a molecular‐targeted therapy (Mosenson et al. 2012). HSP70i_Q435A_ administered via biolistic administration of plasmid DNA into vitiligo‐prone, T cell receptor transgenic mice led to significantly decreased depigmentation by inhibiting DC activation (Mosenson et al. 2013).

In this study, we questioned whether disease progression and long‐term treatment outcomes in response to HSP70i_Q435A_ differed according to sex. Exhibiting the same transgenes responsible for progressive depigmentation, we questioned whether males and females would differentially depigment over time and whether disease progression is impacted by treatment with HSP70i_Q435A_. To this end, we measured depigmentation in h3TA2 mice of different ages and stages of disease after treatment with HSP70i_Q435A_‐encoding DNA and maintained the animals for up to 46 weeks of age. Mouse sera were analyzed for anti‐HSP70 serum titers, and cutaneous immune infiltration was evaluated by immunohistology. T cell activity was measured by ELISA and ELISPOT analysis of IFN‐γ and IL‐10 secreting T cells. These studies provide awareness of mechanisms defining disease severity and responses to treatment according to sex and can serve to suggest adjustments to patient treatment plans as needed. The outcomes can also be helpful to suggest better approaches to clinical trial design for vitiligo treatment.

## 2 Results

### 2.1 Depigmentation Develops More Extensively in Female Vitiligo‐Prone Mice

The h3TA2 mice express a tyrosinase‐reactive TCR derived from human TIL1383I (Nishimura et al. 1999), reactive with peptide TYR_367‐377_ derived from tyrosinase and presented in the context of HLA‐A2, causing them to spontaneously depigment over time. Scans of the mice before treatment at various ages (Figure 1) demonstrate significantly more depigmentation in females as compared to males at each initiation time point (Figure 2A). Two‐way ANOVA values for sex (*p* = 2.56e−07) and time (*p* < 2e−16) showed significant differences for both parameters, with 1.63‐fold greater depigmentation in females than in males and 6.40‐fold increased natural depigmentation in mice treated at 36 weeks compared to those at 5 weeks. Example mice at 5, 6, 8, 12, 20, and 36 weeks before treatment show progressive depigmentation as initial treatment, with females depigmenting more readily and extensively (Figure 2B).

**FIGURE 1:**
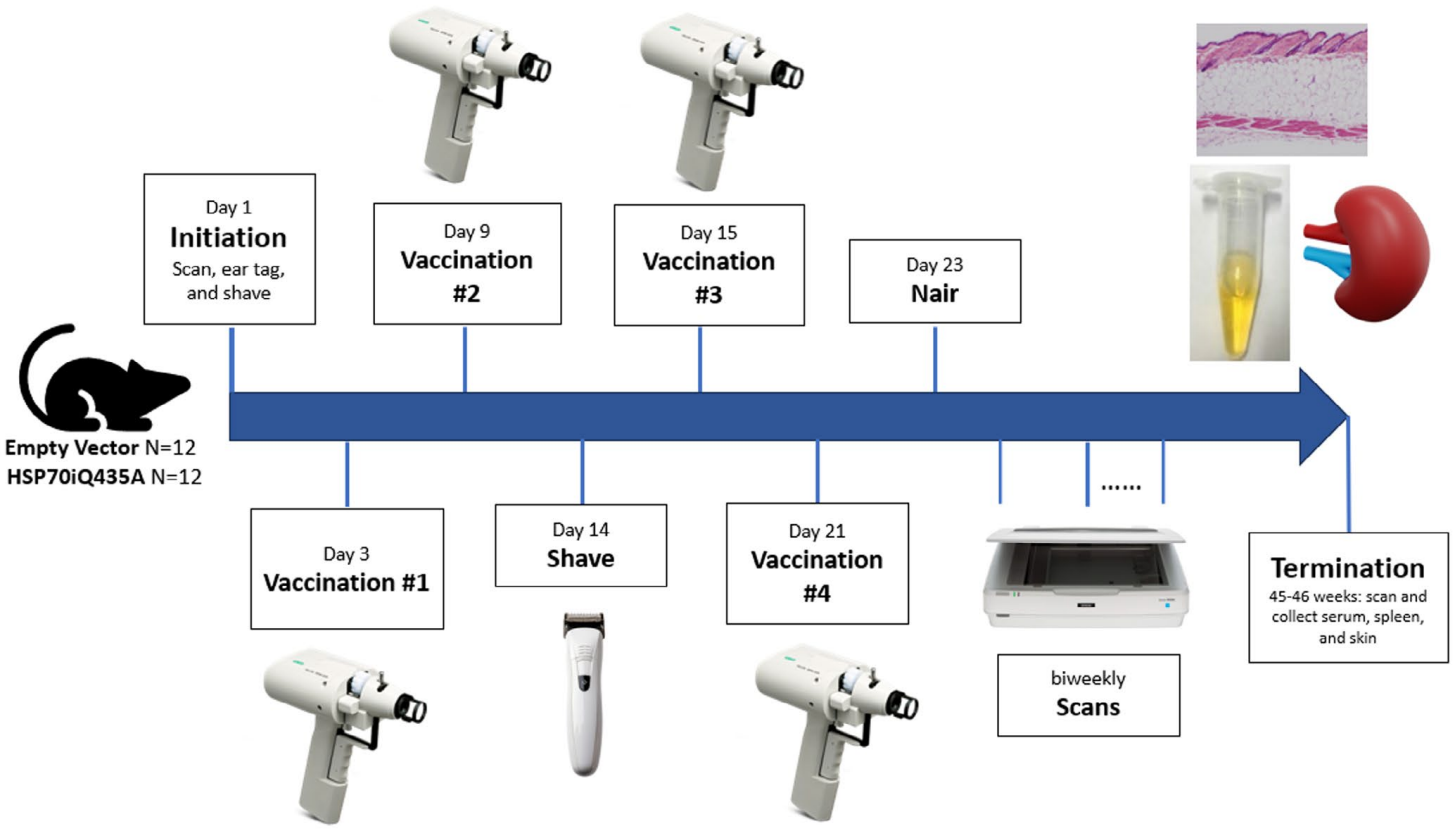
Treatment schematic. Groups of 12 mice were entered into experiments starting at ages 5, 6, 8, 12, 20, and 36 weeks and received biolistic gene gun vaccination with either HSP70iQ435A or empty vector DNA. Mice were shaved and placed under anesthesia for a total of four treatments, 6 days apart. The mice were then scanned for depigmentation every 2 weeks until they reached 45–46 weeks of age. Spleens were collected as well as serum and skin samples.

**FIGURE 2:**
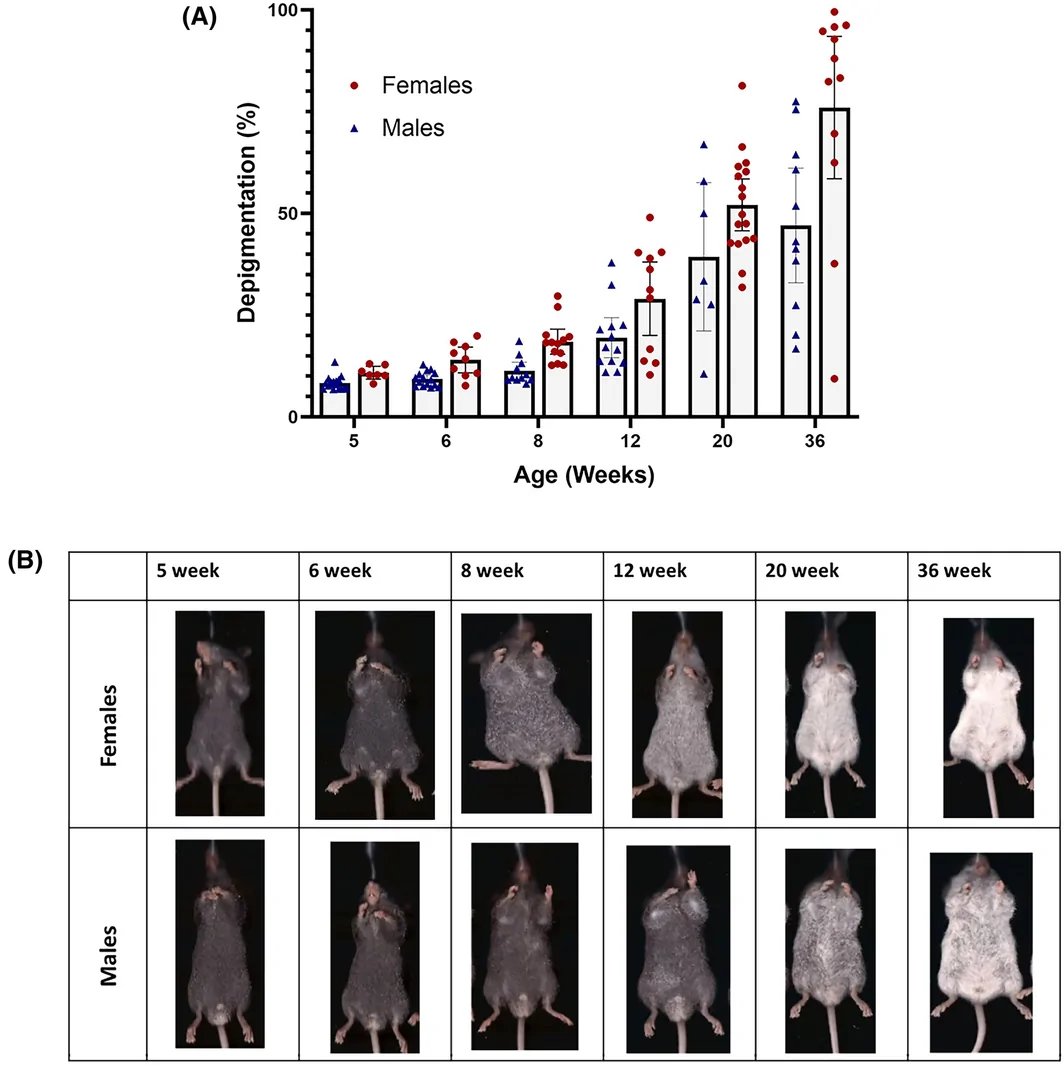
Depigmentation develops more extensively in female vitiligo‐prone mice. (A) Depigmentation in h3TA2 mice was measured prior to treatment with HSP70i_Q435A_ in mice of various ages and plotted by sex. Female mice depigmented 1.89‐fold more overall than male mice before treatment (***p = 9.82e−6). Depigmentation compared by two‐way ANOVA for sex (***p = 2.56e−07) and time (***p < 2e−16) showed significant differences for both parameters. (B) Representative h3TA2 mice at various time points without treatment. Female (*N*) = 70, Male (*N*) = 74.

### 2.2 Females Depigment More Than Males, While Both Benefit Equally From Treatment

The average depigmentation was calculated from all time points after treatment for empty vector (EV)‐treated males, HSP70i_Q435A_‐treated males, EV‐treated females, and HSP70i_Q435A_‐treated females. Overall, depigmentation of females was reduced by 13% (*p* = 3.60e−14) in response to HSP70i_Q435A_ over the full period of evaluation as measured by two‐way ANOVA, while the same treatment reduced the depigmentation of males by 15.74% (*p* = 2.37e−8) (Figure 3A). While males depigmented less overall, both sexes treated with HSP70i_Q435A_ received similar benefit from the treatment, and interestingly, this treatment benefit remained visible over the full period of evaluation (Figure 3B).

**FIGURE 3:**
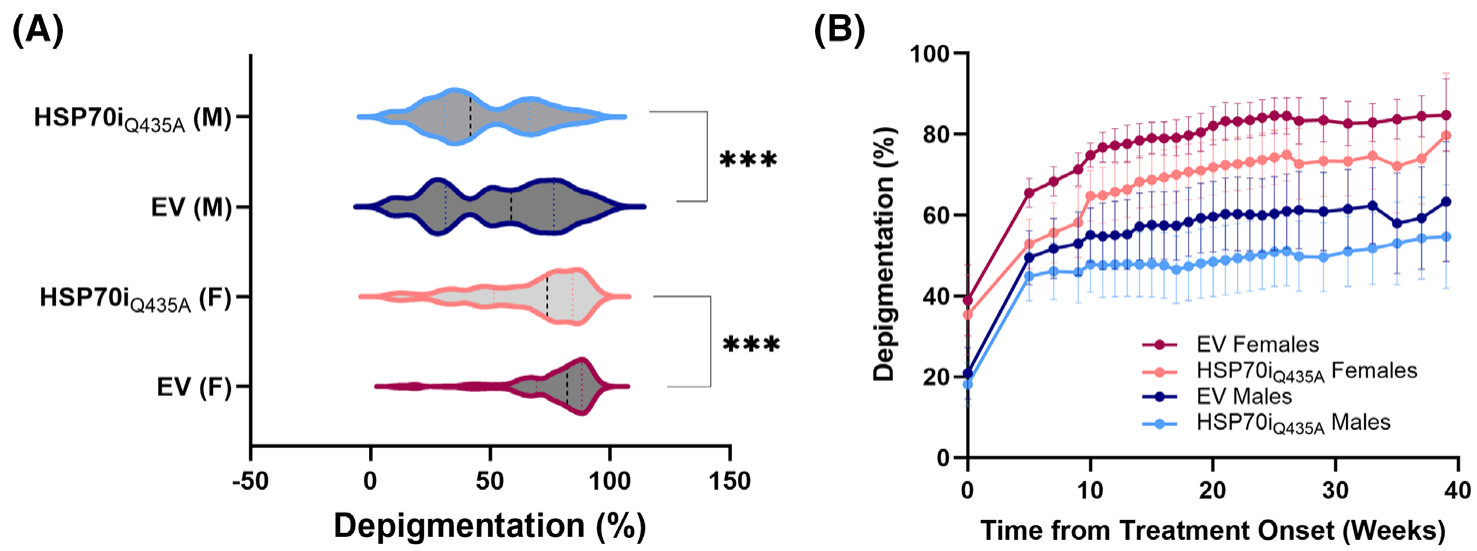
Females depigment more than males while both benefit equally from treatment. (A) The average percent depigmentation when the pelage returned was significantly greater in females than males. EV and HSP70iQ435A‐treated females were of 76.59% and 66.66% depigmented (***p = 3.60e−14), while EV and HSP70i_Q435A_‐treated males were 55.30% and 47.23% depigmented (***p = 2.37e−8), respectively. Thus, males and females benefited equally from HSP70i_Q435A_ treatment. (B) Groups maintained differences in depigmentation over time after treatment. EV‐treated females (*N*) = 33, HSP70i_Q435A_‐treated females (*N*) = 37, EV‐treated males (*N*) = 39, HSP70i_Q435A_‐treated males (*N*) = 35.

### 2.3 The Timing of Greatest Treatment Benefit Is Time‐ and Sex‐ Dependent

Mice were treated at 5, 6, 8, 12, 20, or 36 weeks of age, wherein mice treated in earlier stages of disease exhibited a trend towards greater treatment benefit compared to mice treated later (Figure 4A). In particular, mice that received treatment with HSP70i_Q435A_ at 5, 6, 8, and 12 weeks displayed a significant benefit to treatment at 11 (*p* = 0.04), 15 (*p* = 0.03), 17 (*p* = 0.04), 19 (*p* = 0.03), 21 (*p* = 0.03), and 23 (*p* = 0.05) weeks after treatment (Figure 4B). This significant treatment difference was not seen in later treatment groups at 20 and 36 weeks of age.

**FIGURE 4:**
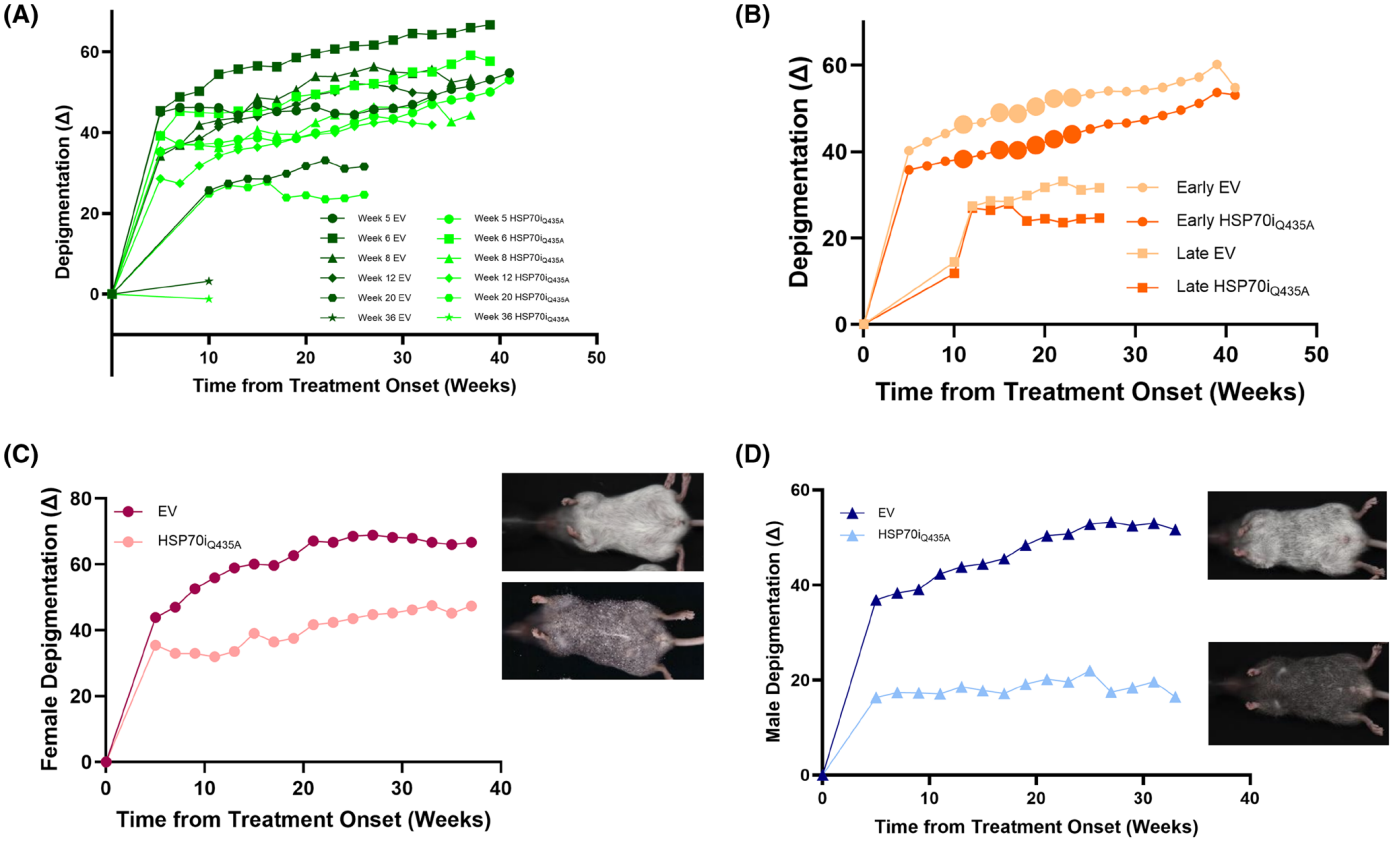
The timing of greatest treatment benefit is sex‐dependent. (A) Mice treated closer to disease onset exhibited a trend towards greater benefit from treatment than older mice with more extensive disease. (B) Mice combined from weeks 5, 6, 8, and 12 displayed a significant treatment benefit during weeks 11 (*p = 0.04), 15 (*p = 0.03), 17 (*p = 0.04), 19 (*p = 0.03), 21 (*p = 0.03), and 23 (*p = 0.05) after treatment. The treatment does not impact depigmentation for mice treated at later stages (20 and 36 weeks). Enlarged data points represent significant treatment benefit for the combined data. EV‐treated females (*N*) = 33, HSP70i_Q435A_‐treated females (*N*) = 37, EV‐treated males (*N*) = 39, HSP70i_Q435A_‐treated males (*N*) = 35. (C) In females, greatest benefit from HSP70i_Q435A_ treatment was observed when initiated at 8 weeks of age, with a significant difference between treatment group and screening weeks (***p < 2e−16, 2‐way ANOVA). HSP70i_Q435A_‐treated (*N*) = 7, EV‐treated (*N*) = 6. (D) In males, greatest treatment benefit was observed when initiated at 12 weeks of age, with a significant difference between treatment group and screening weeks (***p < 2e−16, 2‐way ANOVA). HSP70i_Q435A_‐treated (*N*) = 6, EV‐treated (*N*) = 7. EV = empty vector DNA.

The time of treatment initiation that resulted in mice benefitting most differs between males and females. Females demonstrated the greatest benefit from treatment at 8 weeks of age (Figure 4C). Two‐way ANOVA analysis comparing treated to EV‐exposed females revealed a 21.42% reduction in depigmentation over all time points (*p* < 2e−16), while the treatment effect was also impacted by time. For males, the greatest treatment benefit occurred at 12 weeks of age, with two‐way ANOVA analysis demonstrating significantly reduced depigmentation by 28.58% (*p* < 2e−16), similarly influenced by the timing of measurement (*p* < 2e−16) (Figure 4D).

### 2.4 Depigmentation Correlates With Loss of Melanocytes and Skin Infiltration by T Cells

Compared to a pigmented C57BL/6 mouse, in immuno‐stained skin sections of mice selected for the treatment time point of greatest treatment benefit, vitiligo can be observed (Figure 5). Loss of melanocytes and T cell infiltration of hair follicles are evident in all samples of vitiligo mouse models at end point, showing that the mice develop true vitiligo, while melanocytes are abundant in the hair follicles of control skin. Differences in melanocyte‐containing hair follicle abundance were not significant between males and females, at 26.81% and 24.52%, respectively (*p* = 0.73).

**FIGURE 5:**
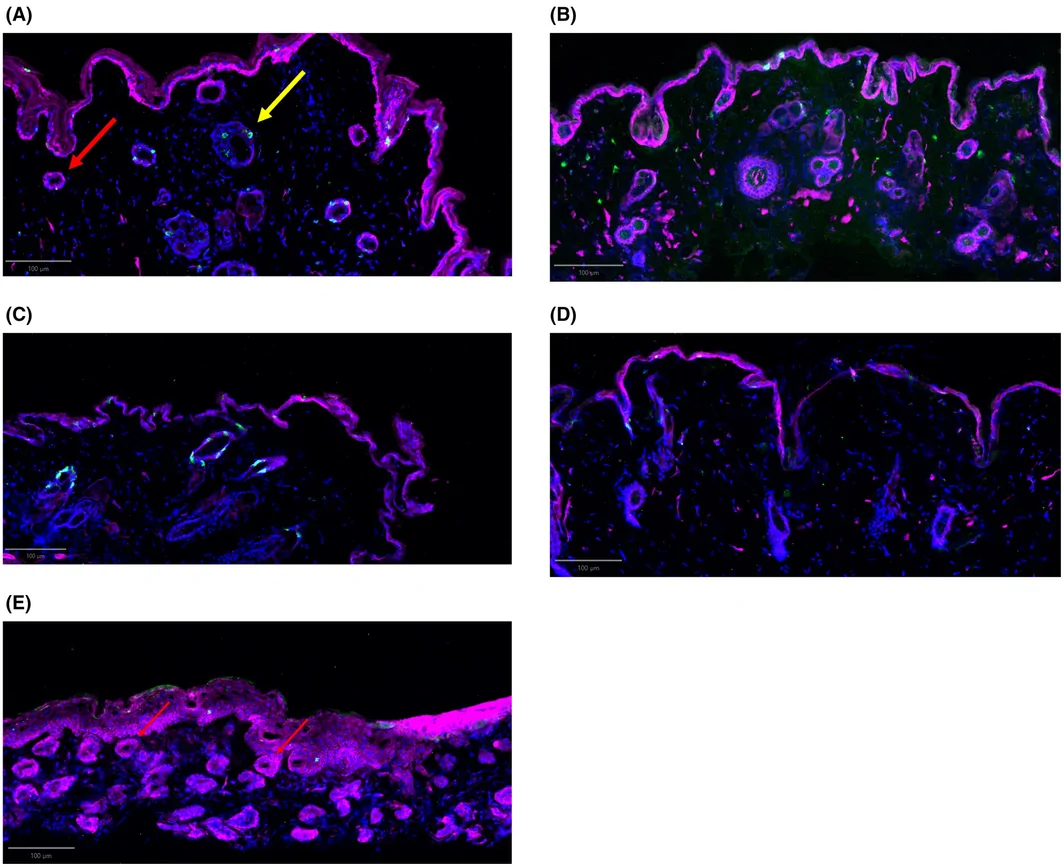
Depigmentation correlates with loss of melanocytes and skin infiltration by T cells. (A) Sample of skin staining in a week 12, HSP70iQ435A‐treated male mouse at end point. Melanocytes are tagged with an AF750‐labeled antibody (magenta), and T cells with an AF647‐labeled antibody (green). The yellow arrow indicates a hair follicle with T cell infiltration and no melanocytes, while the red arrow indicates a hair follicle that still contains melanocytes. Scalebars shown at 100 μm. (B) Sample of an HSP70i_Q435A_‐treated female mouse from the group that started treatment at week 8 is shown at end point. (C) Sample of an EV‐treated female mouse that started treatment at week 8 is shown at endpoint. (D) Sample of an EV‐treated male mouse that started treatment at week 12 is shown at end point. (E) Sample of a C57BL/6 control mouse.

### 2.5 Differential Responses by Male and Female T Cells Are Defined by Different Levels of IFN‐γ Secretion and Suppressive T Cell Populations

When analyzed results of all available splenocytes with combined HSP70i_Q435A_ and EV‐treated splenocytes separated by sex, secretion of IFN‐γ by unstimulated splenocytes from both male and female mice was minimal. However, female mice consistently expressed higher amounts of IFN‐γ in response to stimulation with 3 μg/mL cognate mouse tyrosinase peptide (mTYR) at an average of 40.00 ng per million cells in females and 14.45 ng per million cells in males (*p* = 0.048) (Figure 6A). The number of cells producing IFN‐γ in response to stimulation with 3 μg/mL mTYR was also greater among females at 3168 per 10^6^ cells, versus 819.2 per 10^6^ cells among males (*p* = 0.024) (Figure 6B). Sample ELISpot wells are shown (Figure 6C).

**FIGURE 6:**
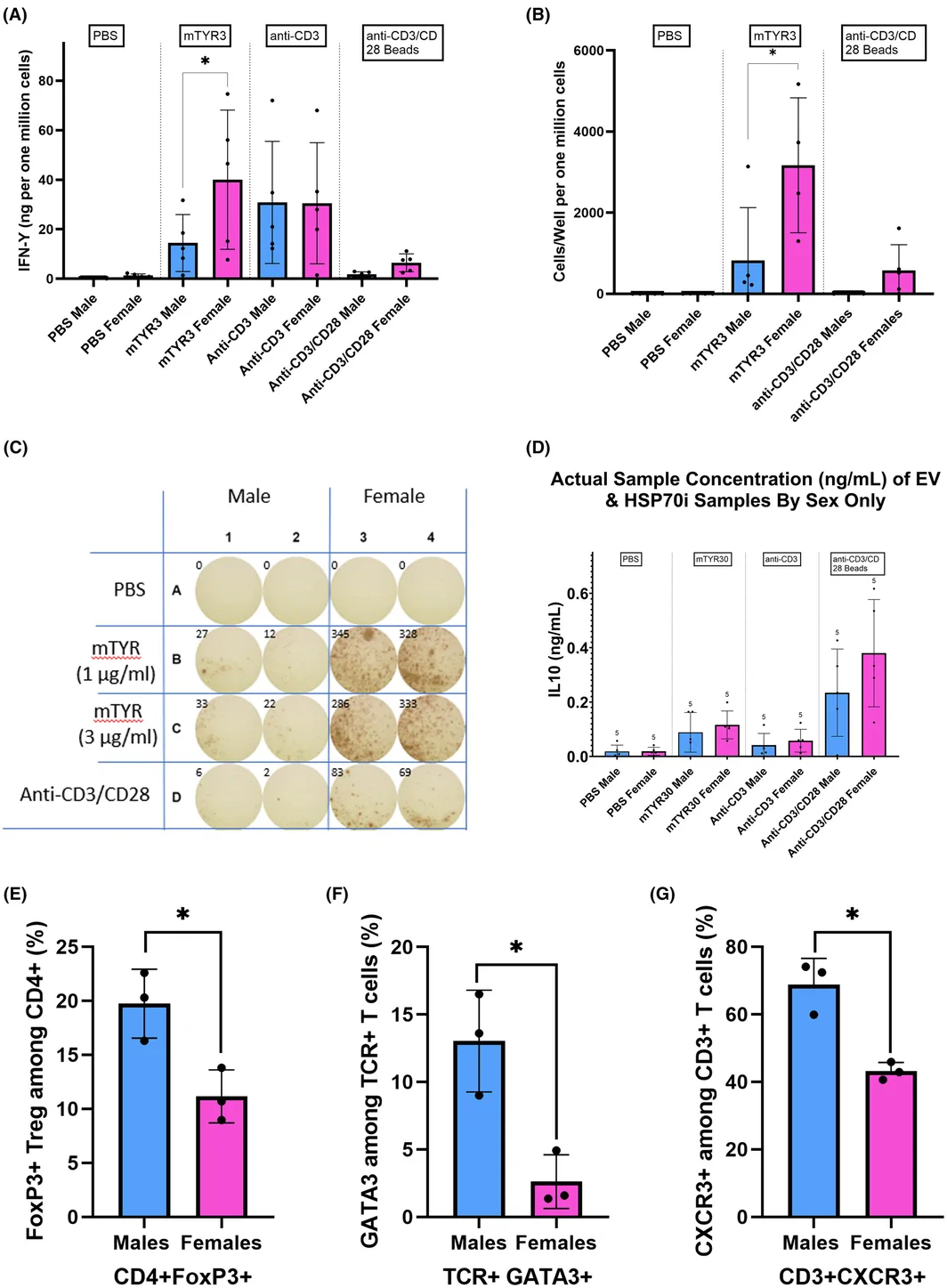
Differential responses by male and female T cells are defined by different levels of T cell polyfunctionality. (A) Measured by ELISA, IFN‐γ production by untreated splenocytes is minimal, while TCR stimulation with cognate mTYR peptide produced greater IFN‐γ release from female than male splenocytes (*p = 0.048). (B) IL‐10 production was more apparent after general stimulation of splenocytes by anti‐CD3/CD28 beads. (C) By ELISpot, when stimulated with 3 ug/ml of mTYR, the number of cells secreting IFN‐γ is significantly greater among splenocytes from female mice (*p = 0.024). (D) Sample ELISpot wells from a male and a female EV‐treated mouse that started treatment at 12 weeks. Differential abundance of (E) CD4 + FoxP3+ Tregs (*q = 0.04), (F) TCR + GATA3+ Type 2 antigen‐specific T cells (*q = 0.04) and (G) CD3 + CXCR3+ T cells (*q = 0.04) among males is also shown.

Overall, secretion of IL‐10 was produced primarily following general stimulation from anti‐CD3/CD28 beads. Female cells produced 0.38 ng/mL on average, while male cells produced 0.23 ng/mL, but the two were not significantly different (Figure 6D).

In a separate experiment performed using splenocytes of either sex from younger mice, the observed changes are associated with a significantly larger population of CD4+ Treg (*q* = 0.04) and Type 2 antigen‐specific T cells (*q* = 0.04) in males, while Type 1 and Type 17 antigen‐specific T cells were not significantly different in abundance. CXCR3+ T cells were likewise more abundant in males (*q* = 0.04), but neither the CD4‐CD8‐ antigen‐specific, the CD4+ nor the CD8+ populations alone met criteria for a significant difference among both sexes.

### 2.6 Vaccine‐Induced Anti‐HSP70i Serum Titers Minimally Wane Over Time

Anti‐HSP70i antibody titers, known to protect from melanoma development, were measured in at least 3 serum samples for both treatment and control groups for mice treated either before or after 20 weeks of age, representing the starting point of accelerated depigmentation (Figure 7) (Jaishankar et al. 2021). Mice treated more recently, i.e., after 20 weeks, exhibited on average a 13.11‐fold elevation in anti‐HSP70i titers in comparison to EV titers (*p* = 0.002). Remarkably, even in mice treated more than 25 weeks prior, anti‐HSP70i antibody titers were still increased 5.85‐fold over EV titers (*p* = 0.003), demonstrating the long‐lasting impact of this gene gun treatment. Control mice treated with EV DNA generated less than 500 ng/mL across all groups.

**FIGURE 7:**
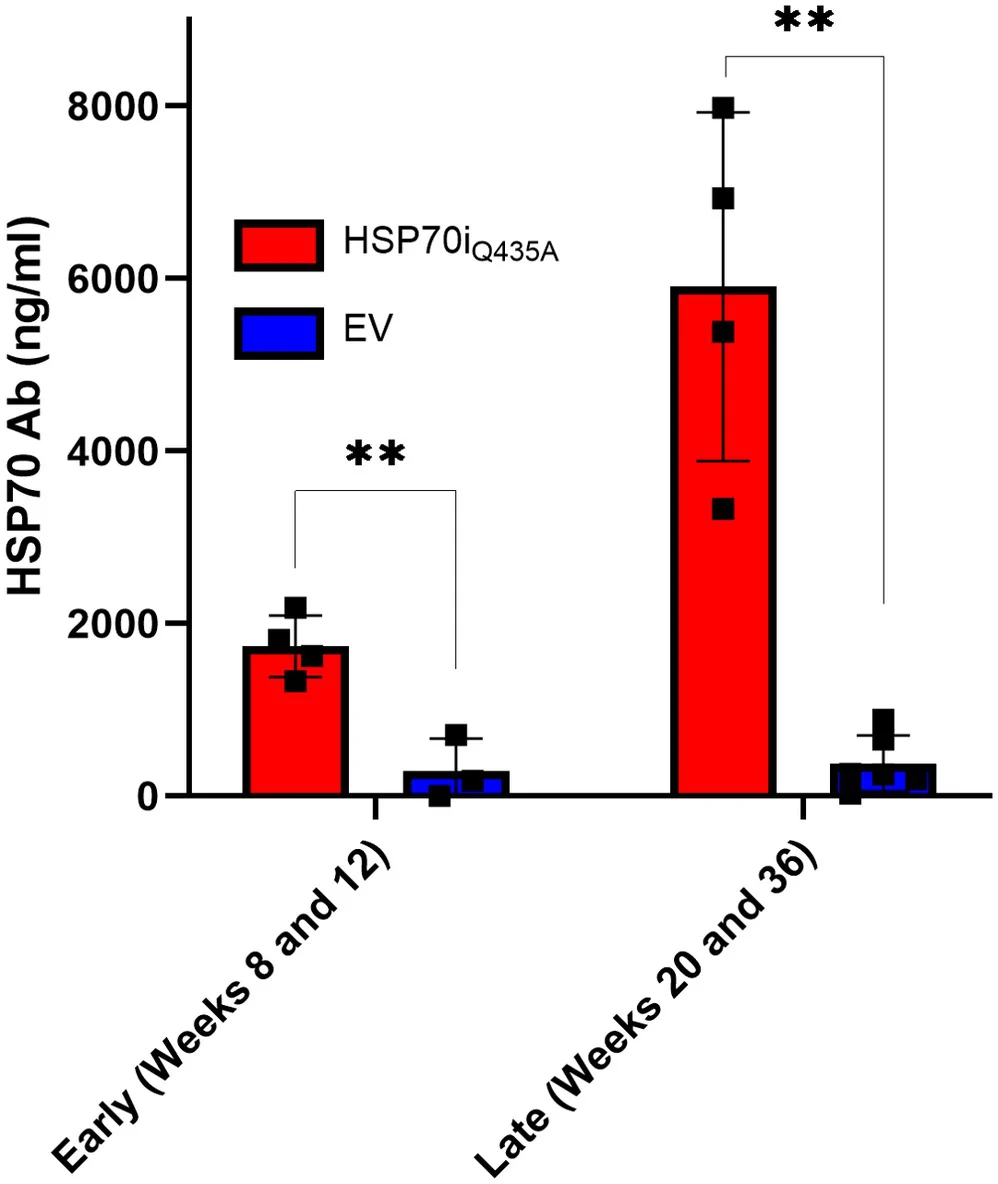
Vaccine‐induced anti‐HSP70 serum titers minimally wane over time. Anti‐HSP70 Ab titers were measured for treatment groups starting treatment early (max 12 weeks of age) or late (starting treatment at 20 weeks of age or more) compared to untreated mice. Titers were significantly higher in groups treated with HSP70i_Q435A_ compared to EV in both the early (**p = 0.003) and late (**p = 0.002) groups. Early, HSP70i_Q435A_‐treated (*N*) = 4; Early, EV‐treated (*N*) = 3; Late, HSP70i_Q435A_‐treated (*N*) = 5; Late, EV‐treated (*N*) = 6.

## 3 Materials and Methods

### 3.1 Mice

Vitiligo‐prone h3TA2 mice were bred in house at Northwestern by crossing h3T transgenic animals with HLA‐A2 transgenic mice purchased from the Jackson Laboratory (RRID: IMSR_JAX:004191). Resulting mice express the human TIL1383I tyrosinase‐reactive TCR developed from MHC Class I restricted CD4^+^ T cells (Mehrotra et al. 2012). These mice spontaneously develop vitiligo starting from around 3–4 weeks of age (Mehrotra et al. 2012). Experiments included groups of 12 mice entering the experiments at either 5, 6, 8, 12, 20, or 36 weeks of age. Groups contained varying numbers of male and female mice, but each group had at least three mice of each sex. All studies involving mice were approved by the Northwestern Institutional Animal Care and Use Committee and were in compliance with the US National Research Council's Guide for the Care and Use of Laboratory Animals and the US Public Health Service's Policy on Humane Care and Use of Laboratory Animals.

### 3.2 Gene Gun Vaccination

To generate bullets, plasmid DNA was precipitated onto 1 μm gold beads (BioRad #1652263) in spermidine and CaCl_2_ solution and washed with 100% ethanol. The DNA‐coated beads were introduced into Tefzel tubing (BioRad #1652441) and dried under nitrogen. Individual bullets contained 1.4 μg of HSP70i_Q435A_‐encoding or empty vector (control) DNA. Bullets were maintained under vacuum and used within 21 days. Mice were placed under anesthesia for exposure to four treatments, 6 days apart by biolistic gene gun vaccination using the Helios gene gun system (BioRad #1652411) onto Nair‐treated hairless ventral skin, introducing total doses of 5.6 μg of DNA per week under helium pressure.

### 3.3 Evaluating Depigmentation

Depigmentation was evaluated 2 days prior to initial treatment and starting 5 weeks after the final treatment, whenever the pelage had sufficiently regrown. Mice were then scanned every 2 weeks until 45–46 weeks of age, quantifying depigmentation essentially as described. Briefly, mice were anesthetized and placed on a flatbed scanner (Epson #B11B268201) to measure ventral and dorsal depigmentation. Image analysis was performed using Adobe Photoshop software (Denman et al. 2008). The percent depigmentation was evaluated using luminescence determined by the proportion of pixels among > 150,000 evaluated along a continuum of 0% depigmentation, correlating to a fully pigmented pelage, to 100% depigmentation, correlating to a fully depigmented pelage. Depigmentation was plotted over time.

### 3.4 Immunohistology

After euthanasia, mouse skin samples were embedded in optimal cutting temperature (OCT) compound (Sakura Finetek #4583) and snap‐frozen. Cryostat sections of 8 μm were fixed in cold acetone and stored at −20°C until use. Immunostaining was performed on male tissues that were treated at week 12 and female tissues treated at week 8, when animals received best treatment benefits. The experiment included HSP70i_Q435A_ and EV‐treated mice, and pigmented C57BL/6 mouse skin as a control. Tissue sections were dual stained with antibody C9 to DCT (TRP2) and an AF750‐labeled goat anti‐rabbit secondary (Abcam #ab175732), as well as with AF647‐labeled hamster anti‐mouse CD3 antibody clone 145‐2C11 (BioLegend #100322, RRID:AB_389322). Staining was imaged using the Nikon Ti2 widefield microscope in Northwestern's Center for Advanced Microscopy. Stained hair follicles or cells were quantified per length of epidermis as quantified in QuPath (v0.6.0).

### 3.5 ELISpot Assay

Peptide reactive T cells were quantified using ELISPOT kits (BD Bioscience #BDB551849). Briefly, plates were coated with capture anti‐IFN‐γ antibody. Responder splenocytes (1.25 × 10^5^ cells) from treated mice were cultured with T2‐A2 cells (2.5 × 10^4^ cells/wells) with 3 μg/mL mouse tyrosinase peptide FMDGTMSQV (Pi Proteomic) or not. After 24 h of incubation at 37°C in a CO_2_ incubator, plates were evaluated for the development of IFN‐γ spots according to the manufacturer's manual (BD Bioscience).

### 3.6 ELISA on Splenocytes and Sera

Detection of IFN‐γ and IL‐10 release from mouse splenocytes was performed by ELISA. Briefly, responder splenocytes (5 × 10^5^ cells) from treated mice were cultured with T2‐A2 (2.5 × 10^4^ cells/well) in presence of 3–30 μg/mL mouse tyrosinase peptide FMDGTMSQV (Pi Proteomic) or PBS. After 48 h of incubation at 37°C in a CO_2_ incubator, plates were spun down, and culture supernatants were frozen at −80°C until analysis. IFN‐γ and IL‐10 were detected using IFN‐γ (ThermoFisher #88‐7316‐22) and IL‐10 ELISA kits (Mabtech #3430‐1HP‐1) according to the manufacturer's manuals. Also, anti‐HSP70 antibodies were measured in mouse sera to quantify the response to treatment. Serum samples were used to measure anti‐HSP70 antibodies in response to treatment in vivo. High‐sensitivity ELISA test kits (Enzo Life Sciences #ADI‐EKS‐750) were used according to the manufacturer's instructions. Plates pre‐coated with antibodies to inducible HSP70 were incubated with diluted serum collected at end point. For all ELISAs, enzyme‐labeled detection antibodies were used prior to adding to wash plates, and after incubation, plates were washed again prior to adding 3, 3′, 5, 5′‐tetramethylbenzidine (TMB) substrate. A BioTek Synergy H1 microplate reader was used to detect color change at 450 nm and titers were calculated from a standard curve.

### 3.7 FACS Analysis

Transcription factor expression was examined to estimate T cell subset abundance by intracellular staining. Splenocytes were harvested from 6 mice (3 males and 3 females), 12 weeks ±1 week of age. Dead cells were excluded using a Ficoll gradient (MilliporeSigma, #GE17‐1440‐02) and cell viability was confirmed by Trypan Blue exclusion (MilliporeSigma #T8154). Fc receptor blocking among live splenocytes was accomplished by adding 2.4G2 anti‐CD16/CD32 Fc receptor antibody (BD Biosciences, #553140) for prior to staining with antibodies to surface markers SR718‐labeled17A2 Ab to CD3 (Biolegend #1002825), BV650‐labeled GK1.5Ab to CD4 (BioLegend #100469), BV510‐labeled 53‐6.7 Ab to CD8 (BioLegend #100751) and FITC‐labeled S511 Ab to the Vβ12 TCR subunit (ThermoFisher Scientific #3213446) before preparing cells for intracellular staining. Cells were subsequently fixed and permeabilized using a Fix/Perm bufferset (BD Biosciences #560409). The following mAbs to nuclear factors were included: AF647‐labeled MF‐14 to FoxP3 (BioSciences #126408), PerCPCy5.5‐labeled 4B10 Ab to T‐bet (BD Biosciences #644806), and BV421‐labeled 16E10A23 to GATA3 (Biolegend #653814) and PE‐labeled AFKJS‐9 Ab to RORγt (ThermoFisher Scientific #12‐6988‐82). To examine surface CXCR3 expression, a separate sample from each mouse was subjected to Zombie NIR solution (BioLegend #423105) and washed, followed by surface staining by AF488‐labeled 17A2 Ab to CD3 (BioLegend, #100210), BV421‐labeled V4 Ab to CD4 (BioLegend #100443), AF647‐labeled 53–6.7 Ab to CD8 (BioLegend #100734) and BV480‐labeled CXCR3‐173 Ab to CXCR3 (ThermoFisher Scientific #414‐1831‐82). Samples were analyzed on Cytek Aurora flow cytometry instrument (Cytek Biosciences Inc. #N7‐00090). Data and figures were prepared using FlowJo (Waters Corporation version#10.1).

### 3.8 Statistical Analysis

Statistical analyses were performed in GraphPad Prism (version 9.3, GraphPad Software) or using R (version 4.4.1) program for statistical computing (https://www.R‐project.org) as specified in the figure legends. Significance was set at *p* < 0.05. Briefly, the dataset was first restricted to observations corresponding to animals with a starting age of 5 weeks in relative weeks. For descriptive visualization, individual subject trajectories were plotted using line graphs, and smoothed trend lines were added to illustrate overall temporal patterns. Faceted figures were generated to display trajectories separately by experimental group. To assess the main effects of time and treatment group, a linear mixed‐effects model was fitted with ventral depigmentation percentage as the outcome. The model included time—treated as a categorical variable—treatment group, and a random intercept for subject to account for within‐subject correlation. Marginal means and 95% confidence intervals were estimated using the emmeans package, and model‐based confidence intervals for fixed effects were obtained with the ci.lin function. To compare overall group differences at specific time points, a two‐way ANOVA was performed. For comparisons of depigmentation percentages between two independent groups (e.g., by treatment or sex), two‐sample *t*‐tests were used. Adjustments for multiple comparisons were not applied due to the limited number of statistical tests conducted.

## 4 Discussion

The development of vitiligo is a dynamic process involving cytotoxic T cell destruction of melanocytes. This T cell‐mediated killing of melanocytes is further permitted by a relative lack of regulatory T cells (Tregs) in vitiligo skin, which can be replenished by Treg adoptive transfer (Chatterjee et al. 2014; Le Poole and Mehrotra 2017; Mukhatayev et al. 2020). Our study further enhances this growing understanding by highlighting the interplay of this immunopathology with both the sex and stage of disease to influence disease severity and therapeutic outcome.

Many autoimmune diseases disproportionately affect women, including systemic lupus erythematosus, multiple sclerosis (MS), and rheumatoid arthritis (Whitacre et al. 1999), alopecia areata (Lundin et al. 2014; Thompson et al. 2024), oral lichen planus (Olson et al. 2016), and Vogt‐Koyanagi‐Harada syndrome (Greco et al. 2013). Studies on vitiligo likewise favor a female predominance (Cedercreutz et al. 2010; Zhang et al. 2016). The increased prevalence of vitiligo in women might in part be attributed to social and cosmetic concerns that would lead to earlier detection in women, yet studies aiming to limit confounding bias by measuring prevalence of vitiligo amongst family members reported rates as much as 25% higher in women than in men, reinforcing this female predominance (Cedercreutz et al. 2010). The increased prevalence of autoimmune disease in women has prompted researchers to search for genetic, hormonal, and immunologic explanations for these sex differences. A more thorough understanding of these differences may allow for more timely diagnosis and optimal treatment options for men and women.

While it can be more difficult to clinically study sex differences due to concerns of bias, modeling the disease in mice allows for more controlled comparison. By utilizing vitiligo‐prone mouse models, our study investigated the influence of time and sex in the development and treatment of vitiligo. The h3TA2 mouse model expressed a TIL1383I tyrosinase‐reactive TCR transgene, causing spontaneous depigmentation around 3–4 weeks of age. The spontaneous yet inevitable development of vitiligo allowed us to investigate the progression of disease in male and female mice starting from an equal base pigmentation and propensity to develop disease. Depigmentation naturally developed more extensively in female mice before experimental intervention, indicating a predilection for the female immune system to develop more extensive and rapid disease in vitiligo. Clinically, this suggests that women are diagnosed with vitiligo more frequently due at least in part to a biologically more aggressive disease course.

Current theories investigate hormonal, genetic, and immunologic differences between males and females that could account for this exaggerated response and explain the increased prevalence of autoimmunity among females. Some studies have investigated the effects of estrogen and testosterone on the immunological response. In a murine model of experimental autoimmune encephalomyelitis used to investigate MS, female mice treated with testosterone expressed significantly higher levels of IL‐10 and experienced a less severe disease course in comparison to females treated with placebo (Dalal et al. 1997). Sex hormones may also regulate genes involved in immunity. For example, the AIRE genes, which are involved in central tolerance, are upregulated by testosterone and downregulated by estrogen and progesterone, contributing to increased self‐reactivity in females (Berrih‐Aknin et al. 2018; Dragin et al. 2016; Zhu et al. 2016). Another possible explanation for the protection against autoimmune disease observed in males is an increased presence of Tregs and increased expression of FOXP3, a key suppressive molecule associated with Tregs (Dodd and Menon 2022). Testosterone can increase the expression of FOXP3 (Walecki et al. 2015), and parental imprinting effects of the X chromosome also leads to more FOXP3 expression in males (Voskuhl et al. 2018).

While females depigmented more than males, both experienced a proportionately equal benefit from treatment with HSP70i_Q435A_. Furthermore, we observed a significant treatment benefit for several weeks in mice that were treated at earlier stages of disease, which was not seen in mice treated at later stages. Thus, we propose that prompt treatment leads to more favorable outcomes. Previous studies have described similar results. For example, a study observed favorable therapeutic responses to vitiligo treatment in newer lesions, while older lesions were unresponsive to therapy with tacrolimus and narrow band ultraviolet B (NBUVB) phototherapy (Yang et al. 2010). The timing of greatest treatment benefit was, however, sex dependent, with females benefiting most with treatment at 8 weeks of age while males benefited most at 12 weeks. The explanation again likely lies in a complex interplay of genetic, hormonal, and molecular biology. Though functional, real‐time differences in immune activity are expected in the skin, all animals were maintained up to the same time point after vitiligo development was complete. Therefore, disease activity would not be measurable in the skin. Hence the skin samples taken at end point were less informative to address ongoing T cell activity.

We examined a few of the possible causes of accelerated depigmentation in females by measuring T cell expression of IFN‐γ and IL‐10 from all available splenocytes in response to mouse tyrosinase peptide or anti‐CD3/CD28 beads. While we found females produced significantly greater amounts of IFN‐γ in response to mouse tyrosinase peptide, we also found that female splenocytes had significantly more active T cells producing IFN‐γ by ELISpot assay. This suggests that the exaggerated inflammatory response in females is due primarily to a greater number of T cells, rather than a larger production per cell. The production of IL‐10 was not significantly different among the sexes, suggesting that both were equally engaged in tempering the ongoing immune response (Wei et al. 2019). Measurements of IFN‐γ and IL‐10 were performed on splenocytes taken at end point, when vitiligo had progressed beyond peak period of disease activity and the remarkable difference between males and females was evident. Furthermore, these measurements reflected response potential rather than ongoing immune activity. The increased number of IFN‐γ producing T cells in females, while may not be specific to vitiligo, may be a common mechanism in T cell‐mediated autoimmune disease.

T cells of the immunosuppressive Treg and Type 2 subsets were more abundant among males as also observed in prior reports (Chatterjee et al. 2014; Touni et al. 2025), while T cells overall might be more poised to move toward the skin due to elevated CXCR3 expression in active disease. This applied to T cells regardless of the subset they belonged to. It should be mentioned that CXCR3 expression contributed less to depigmentation than IFN‐γ secretion in h3TA2 mice (Chatterjee et al. 2014).

Notably, we found that responses to HSP70i_Q435A_ were long‐lasting in this aggressive model of vitiligo, suggesting that a short treatment regimen may suffice to limit depigmentation unless a new trigger is encountered. We further observed a robust and sustained antibody response to treatment with HSP70i_Q435A_, even in groups that were treated at younger ages. This indicates a long‐lasting response to treatment as well as conferred protection against melanoma tumor cells (Jaishankar et al. 2021).

A limitation of this study is that the mice were not bred to include equal numbers of males and females in each experimental group, yet stratification of this study by sex proved necessary to adequately process the treatment data. This study emphasizes a difference in vitiligo development between the sexes: female mice depigment to a greater extent more rapidly. We found that this increased disease severity coincided with greater IFN‐γ expression from more T cells in female mice. Finally, this paper supports long lasting responses to HSP70i_Q435A_. These findings can inform future biomarker development or therapies, and we emphasize the importance of stratification by sex in future studies of vitiligo.

## Author Contributions

**Kaitlyn G. O'Donnell:** writing – original draft, writing – review and editing, visualization, investigation, validation, formal analysis. **Zhussipbek Mukhatayev:** methodology, data curation, investigation, supervision, validation, formal analysis. **Hanin Abuzer:** data curation, investigation, formal analysis. **Rohan S. Shivde:** investigation, data curation, formal analysis. **Sriram Saravanan Shanmugavelandy:** data curation. **Octavio K. Bartos:** data curation. **Ellis Kang:** data curation. **Emilia R. Dellacecca:** investigation, data curation, formal analysis. **Dinesh Jaishankar:** investigation. **Surya Pandey:** investigation, formal analysis, resources. **Hee Kap Kang:** data curation, formal analysis, supervision, visualization. **Ziyou Ren:** writing – review and editing, methodology, software, data curation, formal analysis, visualization. **I. Caroline Le Poole:** conceptualization, methodology, supervision, funding acquisition, project administration, formal analysis, writing – review and editing.

## Funding

This work was supported by the National Institutes of Health, P30AR075049 and 2R44AR065886, and by Temprian Therapeutics Inc.

## Conflicts of Interest

I.C.L.P. is the CSO for Temprian Therapeutics Inc., a Northwestern spinoff company launched to enable HSP70i_Q435A_ treatment for immune conditions including vitiligo. Other authors have no conflicts of interest to declare.

## Data Availability

The data that support the findings of this study are available from the corresponding author upon reasonable request.
